# In Vitro and In Vivo Characterization of a Pigeon Paramyxovirus Type 1 Isolated from Domestic Pigeons in Victoria, Australia 2011

**DOI:** 10.3390/v13030429

**Published:** 2021-03-08

**Authors:** Songhua Shan, Kerri Bruce, Vittoria Stevens, Frank Y. K. Wong, Jianning Wang, Dayna Johnson, Deborah Middleton, Kim O’Riley, Sam McCullough, David T. Williams, Jemma Bergfeld

**Affiliations:** 1 CSIRO, Australian Centre for Disease Preparedness, Geelong, VIC 3219, Australia; Kerri.Bruce@csiro.au (K.B.); Vicky.Stevens@csiro.au (V.S.); Frank.Wong@csiro.au (F.Y.K.W.); Jianning.Wang@csiro.au (J.W.); D.Williams@csiro.au (D.T.W.); 2 Deakin University, Waurn Ponds, VIC 3216, Australia; dayna.johnson@deakin.edu.au; 3 Agriculture Victoria, AgriBio, Centre for AgriBiosciences, Bundoora, VIC 3083, Australia; Kim.ORiley@ecodev.vic.gov.au

**Keywords:** pigeon paramyxovirus 1 (PPMV-1), Newcastle disease virus (NDV), avian orthoavulavirus 1 (AOAV-1), avian paramyxovirus 1 (APMV-1), pathogenicity, Australia, pigeon, *Columba livia*

## Abstract

Significant mortalities of racing pigeons occurred in Australia in late 2011 associated with a pigeon paramyxovirus serotype 1 (PPMV-1) infection. The causative agent, designated APMV-1/pigeon/Australia/3/2011 (P/Aus/3/11), was isolated from diagnostic specimens in specific pathogen free (SPF) embryonated eggs and was identified by a Newcastle Disease virus (NDV)-specific RT-PCR and haemagglutination inhibition (HI) test using reference polyclonal antiserum specific for NDV. The P/Aus/3/11 strain was further classified as PPMV-1 using the HI test and monoclonal antibody 617/161 by HI and phylogenetic analysis of the fusion gene sequence. The isolate P/Aus/3/11 had a slow haemagglutin-elution rate and was inactivated within 45 min at 56 °C. Cross HI tests generated an R value of 0.25, indicating a significant antigenic difference between P/Aus/3/11 and NDV V4 isolates. The mean death time (MDT) of SPF eggs infected with the P/Aus/3/11 isolate was 89.2 hr, characteristic of a mesogenic pathotype, consistent with other PPMV-1 strains. The plaque size of the P/Aus/3/11 isolate on chicken embryo fibroblast (CEF) cells was smaller than those of mesogenic and velogenic NDV reference strains, indicating a lower virulence phenotype in vitro and challenge of six-week-old SPF chickens did not induce clinical signs. However, sequence analysis of the fusion protein cleavage site demonstrated an ^112^RRQKRF^117^ motif, which is typical of a velogenic NDV pathotype. Phylogenetic analysis indicated that the P/Aus/3/11 isolate belongs to a distinct subgenotype within class II genotype VI of avian paramyxovirus type 1. This is the first time this genotype has been detected in Australia causing disease in domestic pigeons and is the first time since 2002 that an NDV with potential for virulence has been detected in Australia.

## 1. Introduction

Newcastle disease (ND) is one of the most important transboundary viral diseases of poultry and wild birds worldwide. The causative agent, *avian orthoavulavirus 1*, also known as avian paramyxovirus serotype 1 (APMV-1) and Newcastle disease virus (NDV), has an enveloped virion with a non-segmented, single-stranded, negative-sense RNA genome, and belongs to the genus *Orthoavulavirus* of the *Paramyxoviridae* family [1]. The NDV genome is approximately 15 kb and comprises six genes encoding six structural proteins (nucleoprotein [N gene], phosphoprotein [P gene]), matrix [M gene], fusion [F gene], hemagglutinin-neuraminidase [HN gene], and the RNA-dependent RNA polymerase [L gene]) [2]. The amino acid sequence at the protease cleavage site of the fusion protein is known to be a major determinant of NDV virulence [3]. However, in vitro studies have demonstrated that five other genes also contribute to the overall pathogenicity of NDV [4].

NDV has been reported to infect over 200 species of birds, but the severity of disease varies with both host (species, age and immune status) and virus strain (pathotype, dosage and route of infection) [4,5,6]. Based on the severity of disease in chickens, NDV strains are categorized into three main pathotypes, lentogenic (avirulent), mesogenic (moderately virulent) and velogenic (virulent) [7]. Phylogenetically, NDV is classified into two distinct classes, class I and II, all within a single serotype [8]. Class I viruses are almost entirely avirulent and are commonly isolated from waterfowl [9]. Class II viruses comprise the vast majority of the sequenced NDVs, including viruses virulent for poultry across 21 genotypes [10].

Pigeon paramyxovirus type 1 (PPMV-1) is an antigenically variant genotype of NDV with a unique monoclonal antibody (MAb) binding profile [11]. Since first emerging in the Middle East in the late 1970s, it has spread throughout Europe and is now found worldwide [12].

In Australia, outbreaks of disease associated with PPMV-1 were first detected in August 2011, in the state of Victoria. The clinical signs observed in these pigeons included high mortalities (50–100%), along with gastrointestinal and neurological signs. The disease quickly spread to affect domestic pigeons (hobby and fancy pigeons) and feral rock pigeons (*Columba livia*). A spotted turtle dove (*Streptopelia chinensis*) and a collared sparrowhawk (*Accipter cirrocephalus*) were also infected [13].

PPMV-1 has been attributed to a number of outbreaks of ND in chickens and other species [14] and there was a concern that extensive and widespread outbreaks of ND in pigeons might affect the Australian poultry industry and native wild avian species. Whilst the pathogenicity of PPMV-1 for different host species can be variable, a number of studies demonstrated that the virulence of PPMV-1 can increase the following passages through chickens [6,15]. The objective of this study was to investigate the biological, antigenic and genetic properties of the P/Aus/3/11 isolate and undertake both in vitro and in vivo assessments of pathogenicity. This information could then be used to better understand the risk that this virus posed to poultry and to inform management decisions.

## 2. Materials and Methods

### 2.1. Ethics Statement

Animal work was conducted with the approval of the CSIRO Australian Centre for Disease Preparedness (ACDP) Animal Ethics Committee (application number AEC 1498). All procedures were conducted according to the guidelines of the National Health and Medical Research Council as described in the Australian code for the care and use of animals for scientific purposes [16].

### 2.2. Virus Isolation and Identification

Specific pathogen-free (SPF) embryonated chicken eggs (ECEs) were provided by Australian SPF Services Pty Ltd. (Woodend, Australia). ECEs were used for virus isolation, sub-culture and titration. Chicken embryo fibroblast (CEF) cells were prepared from 9–10-day-old SPF embryonated eggs as described previously [17].

Virus isolation from oropharyngeal and cloacal swabs was conducted by inoculation of 9-day-old SPF ECEs via the allantoic cavity. Allantoic fluid that tested positive for haemagglutination was further tested by HI, as described below. Harvested allantoic fluid was aliquoted and stored at −80 °C for subsequent characterisation and use in for chicken challenge trials. The infectious titre of the virus stock was determined by titration in embryonated chicken eggs and the 50% egg infectious dose (EID_50_)/mL was calculated according to the method of Reed and Muench [18]. The prototype viral isolate was obtained from a pigeon cloacal swab and was designated APMV-1/pigeon/Australia/3/2011 (herein referred to as P/Aus/3/11). Chicken antiserum to this isolate was subsequently prepared in SPF chickens at ACDP.

### 2.3. Hemagglutination (HA) and Hemagglutination Inhibition (HI) Tests

Specific avian influenza (H1–H16) and avian paramyxovirus (APMV) (types 1–4 and 6–10) reference stock antigens and antisera (full list of reagents available on request) were prepared in SPF chicken embryos and chickens, respectively, at ACDP using procedures previously described [5]. NDV-specific MAbs 617/161, raised against the APMV-1/Pigeon/Engl/617/83 isolate, and U85, raised against the Ulster 2C isolate, were kindly provided by Dr Ruth Manvell, Veterinary Laboratories Agency, Weybridge, UK.

HA and HI tests were conducted by conventional microtiter methods [5]. Four HA units of each test antigen were tested for reactivity with a panel of avian influenza (AI) and avian paramyxovirus (APMV type 1–4, 6–10) reference antisera as well as the MAbs 617/161 and U85 in HI assays (King 1996). The antigenic relationship between an index virus and other representative isolates was determined from the HI-titre ratios between P/Aus/3/11 and NDV V4 isolate using the Archetti’s formula [19].

### 2.4. Elution Rate and Hemagglutinin Thermostability

The haemagglutinin-elution pattern of the isolate was determined according to previously described methods [20]. Each strain was performed in triplicate in 96-well HA plates and held at 4 °C whilst haemagglutination and elution patterns were observed. At the end of 24 h, the test plate was shaken, the contents resuspended and the test read 2 h later. Red blood cell elution occurring at 24 h or later is recorded as a slow elution time, while elution prior to 24 h is recorded as a fast elution time. The hemagglutinin thermostability of the virus at 56 °C was measured as previously described [21]. Thermostability was reported as the period of persistence of agglutination of chicken erythrocytes within a selected period.

### 2.5. Plaque Formation

Infective allantoic fluid was inoculated onto CEFs and maintained in Minimum Essential Medium (Gibco, Waltham, MA, USA) with and without the addition of 0.1 µg/mL trypsin to determine trypsin dependency for replication [17,22]. CEF cultures were inoculated with ten-fold dilutions of the P/Aus/3/11 isolate and representative NDV strains in 6-well plates (Nunclon). CEF monolayers were washed after 1 hr absorption at 37 °C and overlaid with 2 mL/well of 1.5% (*w*/*v*) carboxymethyl-cellulose (CMC) (Sigma, St. Louis, MO, USA). The cultures were incubated for 6 days at 37 °C in a humidified CO_2_ incubator and plaques visualised by fixing and staining overnight with 0.1% (*w*/*v*) methylene blue in 4% (*v*/*v*) formaldehyde solution. Following washing and drying, plaque diameters were measured directly from the 6-well plates by the selection of discrete and predominant plaques with a millimeter ruler.

### 2.6. Pathogenicity Tests

Mean death time (MDT) in eggs and experimental infections of chickens were used to assess the pathogenicity of P/Aus/3/11. The MDT was determined using SPF chicken embryos using previously described methods [23]. Then, 0.1 mL of each virus dilution (10^−6^ to 10^−9^) were inoculated into the allantoic cavity of five 9–10-day-old embryonated SPF chicken eggs. All eggs were then incubated at 37 °C and examined twice daily for 5 days. The minimum lethal dose is the highest virus dilution that causes all embryos inoculated with that dilution to die. The MDT was determined as the mean time in hours for the minimum lethal dose to kill embryos.

Thirteen, 6-week-old SPF chickens were experimentally infected with P/Aus/3/11. Prior to challenge, serum was collected from each chicken to confirm that birds were serologically negative for NDV antibodies, as determined by HI test. The inoculum was prepared by diluting allantoic fluid in phosphate-buffered saline (PBS) and was administered at a dose of 10^8.7^ EID_50_ in 0.5 mL to each chicken by droplet via the ocular, oral and nasal routes. Chickens were monitored daily for the onset of clinical disease. All birds were swabbed (oral and cloacal) daily and samples were tested by real-time RT-PCR and, for selected PCR-positive swabs, virus isolation in ECEs. Swabs were placed into PBS containing antibiotics at 100 units/mL penicillin (JRH Biosciences, Lenexa, KS, USA), 100 µg/mL streptomycin (Sigma) and 50 µg/mL gentamycin (Sigma). On days 2, 4, 6 and 8 post inoculation (PI), two birds were randomly selected and euthanased for pathological analysis, to attempt to detect any early viral replication in tissues. Euthanasia was performed by cervical dislocation following heart bleed under anaesthesia (ketamine 44 mg/kg, xylazine 8 mg/kg injected intramuscularly). The remaining 5 birds were housed for 3 weeks and then euthanased.

Histological analysis of chicken tissues following infection was performed as described previously [24]. Briefly, tissues were fixed in 10% neutral-buffered formalin for 24 h, processed into paraffin wax, cut and stained using haematoxylin and eosin, along with immunohistochemistry against the NDV nucleoprotein (MAb Q91-6).

### 2.7. RT-PCR and Sequencing

Viral RNA was extracted from virus isolate, P/Aus/3/11, or directly from clinical samples using the MagMAX Viral Isolation Kit (Applied Biosystems, Foster City, CA, USA), as per the manufacturer’s protocol. Real-time RT-PCR was performed to detect viral RNA in swabs using the AgPath-ID One-Step RT-PCR kit (Applied Biosystems, Foster City, CA, USA). Primer and probe sequences targeting the F gene were: 5’-GTCAATCATAATCAAGTTACTCCCAAAT-3’ (forward), 5’- GTAGTCAATGTCCTGTTGTATGCCTC-3’ (reverse) and 5’-FAM–TTTTGCACACGCCT (probe).

Sanger sequencing of complete F-gene sequence from five original specimens (2011-3311 to 2011-3315) was performed. These samples were obtained from the first five properties to be infected with the virus, were all epidemiologically related and were all obtained within a one-month period. In brief, RT-PCR was performed on extracted RNA using the Superscript^®^ III One-step RT-PCR system with platinum^®^ Taq DNA polymerase (Invitrogen, Carlsbad, CA, USA) using previously published primers [25]. DNA was purified following agarose gel electrophoresis using QIAquick gel extraction kit (Qiagen, Hilden, Germany) according to manufacturer’s instructions and then sequenced using the Big Dye terminator sequencing system (Thermofisher, Waltham, MA, USA). Sequencing reactions were run and analysed on a 3130XL genetic analyser (Thermofisher). The full-length fusion genes were checked and consensus sequence compiled using Seqman Pro v.15 in the lasergene software package (DNASTAR, Madison, WI, USA).

Maximum likelihood (ML) phylogenetic trees were constructed using MEGA6 software [26]. The phylogenetic dataset consisted of 189 near-complete NDV F gene sequences (1650 nucleotides in length) from samples in this study along with 125 sequences from the pilot dataset of the recently updated NDV classification system [8] and additional representative NDV genotype VI sequences from Genbank.

Whole genome sequencing was performed using the Illumina MiSeq platform (Illumina, San Diego, CA, USA). cDNA was synthesized using extracted RNA by using cDNA primer: 5’- GTTTCCCAGTAGGTCTCNNNNNNNN-3’, and treated with Klenow DNA polymerase I (Promega) for end repair and PCR amplification was performed using the Roche Expand™ High Fidelity Plus kit (Sigma Aldrich, St. Louis, MO, USA) with primer: cgccGTTTCCCAGTAGGTCTC adapted from Palacios et al. [27]. An Illumina Nextera XT DNA library was prepared from purified PCR products following manufacturer’s instructions. Library DNA was quantified using the Invitrogen Qubit™ ds DNA HS assay (Thermofisher, MA, USA) and the average library size was determined on a bioanalyser using the Agilent high sensitivity DNA kit (Integrated Sciences, Chatswood, NSW, Australia). FASTQ files were obtained following a v2:500 cycle paired end run. Consensus sequence data were obtained following reference mapping in Geneious Pro v.11 (Biomatters, Auckland, New Zealand).

## 3. Results

### 3.1. Virus Isolation and Identification

SPF eggs inoculated with cloacal swabs from diseased or dead pigeons died between 3 to 5 days PI. Allantoic fluid harvested from these eggs showed HA titres ranging from 2^2^ to 2^5^. The P/Aus/3/11 isolate was inhibited by polyclonal antiserum to NDV V4, but not AI or other avian paramyxovirus reference sera. Thus, the P/Aus/3/11 isolate was identified antigenically as avian paramyxovirus serotype 1, and further classified as PPMV-1 using PPMV-1 specific MAb 617/161 in the HI test.

### 3.2. Virus Characterization

Serial passages of the P/Aus/3/11 isolate in SPF ECE at a single dilution of inoculum resulted in increasingly higher numbers of egg deaths following each passage, up until the fifth passage (Table 1). The majority of ECE replicates had an HA titre of 2^7^.

The P/Aus/3/11 isolate showed high levels of cross-reactivity to classical NDV isolates using polyclonal antisera to NDV V4 strain and to P/Aus/3/11 in the HI test (Table 2). However, comparison of cross-reactivity between P/Aus/3/11 and NDV V4 isolates resulted in an R value of 0.25, indicating a significant antigenic difference between these two strains [18]. The NDV-specific Mab U85 reacted at varying titres with all APMV-1 isolates tested, including P/Aus/3/11. However, the PPMV-1 specific Mab 617/161 only reacted with the P/Aus/3/11 isolate.

The haemagglutinin-elution pattern of the P/Aus/3/11 isolate was determined by reading HA titres at 24 hrs and after the resuspension procedure. HA titres were checked until 120 hrs after resuspension. The P/Aus/3/11 isolate had a slow elution rate from chicken red blood cells (Table 3).

The hemagglutinin of the P/Aus/3/11 isolate was considerably thermostable and HA activity was maintained until 45 min incubation at 56 °C, whereas thermostability for APMV-1 isolates B1, Komarov, Herts 33, Texas GB was 5, 15, 25 and 90 min, respectively. The Beaudette C and V4 isolates retained haemagglutinating activity after 120 min incubation (Table 4).

The MDT for the P/Aus/3/11 isolate was 89.2 hr, which is close to the cut off between lentogenic and mesogenic categories of NDVs as per the OIE criteria; that is, lentogenic strains cause death in >90 h, whereas mesogenic strains cause death in 60–90 h.

**Figure 1:**
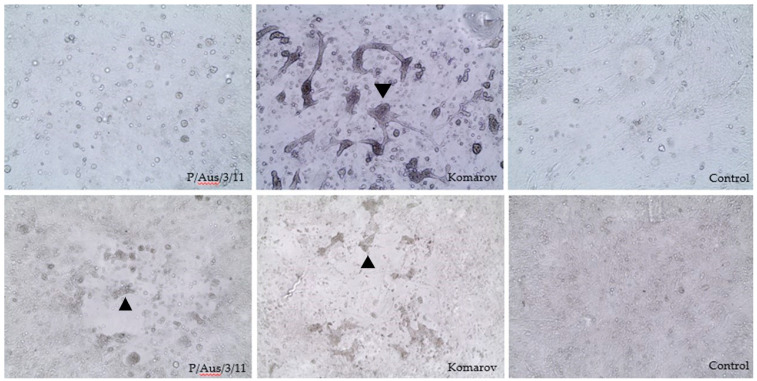
Cytopathic effect (arrowheads) in inoculated chicken embryo fibroblast with P/Aus/3/11, Komorov and CEF control with (bottom three) and without (top three) the addition of carboxymethyl-cellulose in the absence of trypsin (10×).

The P/Aus/3/11 isolate exhibited syncytia in CEFs in the presence of trypsin, compared with single-cell infection without syncytia when trypsin was absent. However, using the CMC overlay, the virus caused syncytium formation without exogeneous trypsin (Figure 1). Although the P/Aus/3/11 isolate may replicate in CEF without the addition of trypsin, five serial passage of virus in CEF did not result in an increase in HA titre, which was similar to the findings with virus growth in chicken embryos.

The P/Aus/3/11 isolate caused plaque formation but grew slowly compared to the reference NDV isolates tested. On day 6 PI, the P/Aus/3/11 isolate produced clear, well-defined plaques of 1–2 mm in diameter, whereas Komarov, Texas GB and Herts produced clear 1.5–2 mm, 2–3.5 mm and 3–4 mm plaques, respectively. The lentogenic strain NDV V4 did not plaque under the experimental conditions used.

When inoculated into chickens, no birds showed any abnormal clinical signs. In addition, there were no lesions detected histologically and no evidence of positive immunohistochemical staining. Virus shedding was assessed by real-time RT-PCR testing of cloacal and choanal swabs. Relatively low levels of shedding from the cloacal route were observed in the infection group, beginning on day 2 PI (2/13 birds) and continuing until day 14 PI (Table 5). However, the percentage of birds shedding on each collection day varied from 0% (day 5 PI) to 60% (day 10 PI). Some birds showed intermittent shedding (# 5, 12, 13), while virus was detected in swabs collected from bird # 10 from day 7 to 12, indicating continuous shedding over this period. Relatively higher levels of virus detection were observed from choanal samples, with all birds testing positive from day 1 to 5 PI (Table 5). Following this, the number of birds that test positive gradually decreased to single birds from day 9 PI. All five birds held until the end of the study had seroconverted with titres ranging between 1:32–1:512/0.05 mL.

### 3.3. Genetic Analysis

A near complete whole genome of the P/Aus/3/11 isolate was sequenced. Nucleotide BLAST analysis showed closest (98.09%) sequence similarity to PPMV-1/Belgium/11-08304/2011-like viruses in Genbank. The amino acid sequence of the F gene of the P/Aus/3/11 isolate showed the multiple basic amino acid motif ^112^RRQKRF^117^ at the cleavage site of F protein, which is classified as virulent. The terminal extension of the HN protein, which has long been suspected to affect virulence [28,29] was found to be zero after the terminal sequence of “KDERV”, consistent with other pigeon paramyxovirus strains and virulent NDVs (Table 6).

In contrast to the genome sequence of the classical vaccine strain V4 (genotype I), the P/Aus/3/11 isolate contains a 6-nt (TCTAAA) insertion in the 3’ UTR of the NP gene at nucleotide 1647. Due to their influence on a range of critical virus functions such as receptor binding, glycosylation sites were examined. Analysis of the F gene revealed six potential glycosylation sites: ^85^N-R-T^87^, ^191^N-N-T^193^, ^366^N-T-S^368^, ^447^N-I-S^449^, ^471^N-N-S^473^ and ^541^N-N-T^543^ with the HN gene containing five potential sites: ^119^N-N-S^121^, ^341^N-N-T^343^, ^433^N-K-T^435^, ^481^N-H-T^483^ and ^508^N-I-S^510^.

**Figure 2:**
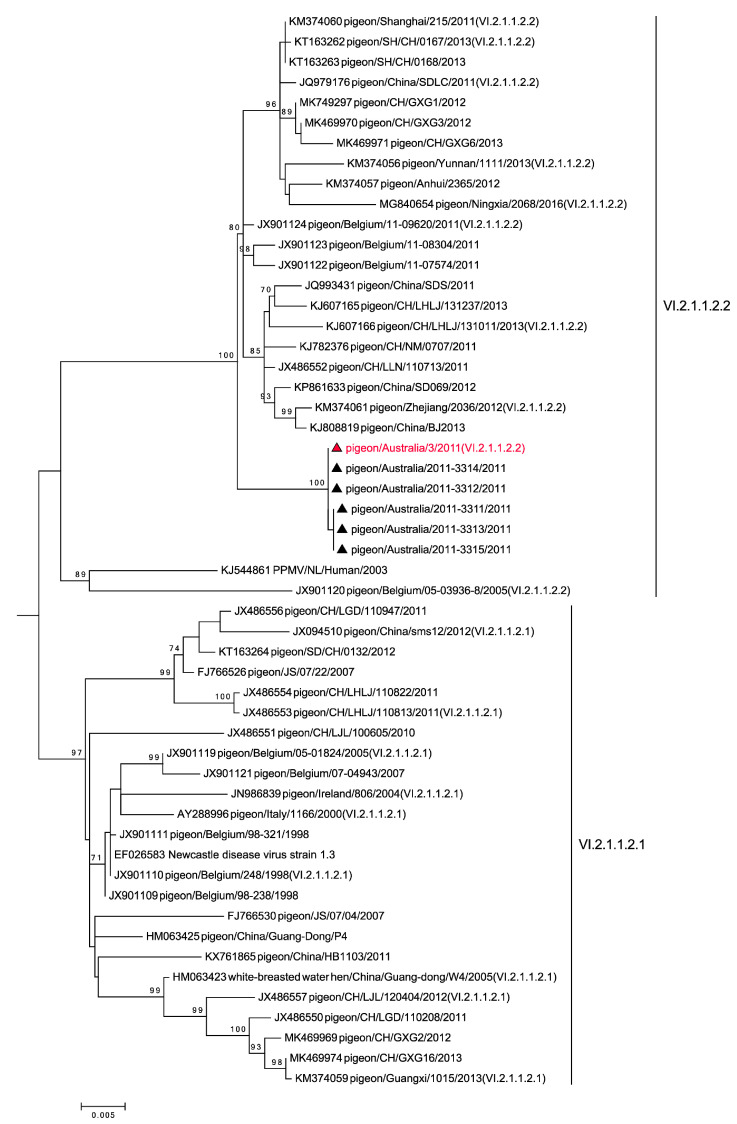
Maximum Likelihood (ML) tree based on the virus fusion protein gene showing genotype VI 2.1.1.2.2 (extracted from Supp. Figure S1 and denoted by vertical lines to the right of tree) with the 2011 Australian PPMV-1 virus samples (denoted by ▲) belonging in genotype VI.2.1.1.2.2. The P/Aus/3/11 isolate is indicated in red. Phylogenetic relationships were inferred using the ML method based on the General Time Reversible model [30]. Horizontal scale bar indicates the number of substitutions per site.

The fusion gene sequences from the 2011 Australian outbreak samples shared 99.9% sequence identity indicating the same virus strain. Phylogenetic analysis of the complete F gene sequences (1650 bp) of the P/Aus/3/11 isolate and the related outbreak samples revealed that they belong to Class II, genotype VI, subgenotype 2.1.1.2.2 (Figure 2), using the recently updated classification system [8]. The P/Aus/3/11 isolate had 98.7% nucleotide identity with APMV-1/pigeon/Germany/5224/2011 and was most similar to contemporary strains circulating in the EU since 2000 (pers. comm. Chad Fuller and Ian Brown, AHVLA, UK). In addition, the P/Aus/3/11 isolate was not related to previous virulent Australian NDVs (Supp. Figure S1), which belong to Class II, genotype I [10]. Virus sequences generated in this study have been deposited into Genbank under accession numbers MN413534 to MN413538 and MN462663 to MN462668.

Analysis of the P/Aus/3/11 genome did not show any notable substitutions in amino acid residues which have been associated with enhanced virulence [31]. In particular, virulence associated substitutions in the L-polymerase (V169E, N1564S) and the phosphoprotein (N37D) were not observed. Instead, the S37 residue in the phosphoprotein, which is the NDV consensus, was present in P/Aus/3/11 and closely related PPMV-1 strains from Belgium and China. Similar to other closely related PPMV-1 strains the P/Aus/3/11 isolated also did not exhibit any of the key F (D72Y, R10M, D114R) and HN (D115S, G362K, E347Q) mutations known to be associated with cross-species adaptation [32,33].

## 4. Discussion

The last outbreaks of Newcastle disease in Australia occurred from 1998–2002, in which virulent ND viruses were found to evolve from endemic, less virulent, lentogenic strains [34]. After eradication of ND in 2002, a vaccination and surveillance program was implemented to minimise the risk associated with ND outbreaks in Australia and this program is still in place today [35]. It was therefore concerning when in 2011, significant mortalities in racing pigeons were detected in association with PPMV-1. As a result, there was a need to further characterise this virus and assess its potential pathogenicity for poultry.

A number of PPMV-1 isolates have been isolated and characterized since ND cases in pigeons were reported in many countries in the 1970s [14,36,37,38]. Although the PPMV-1 isolates comprise a unique subset of NDV based on MAb binding profile, their biological properties frequently overlap the classical NDV isolates with different pathotypes [11]. During this outbreak of PPMV-1 in pigeons in Australia in 2011, the prototype isolate P/Aus/3/11 isolate was biologically, antigenically and genetically characterized. Genetic analysis of F and HN genes showed that the Australian isolate had similar properties to other mesogenic PPMV-1 strains in terms of the F protein cleavage site, the terminal extension of the HN protein and amino acids associated with virulence.

Kommers et al. (2001) stated that not all NDV isolates from pigeons are typical of the variant classified as PPMV-1 [12]. It is well documented that the Mab U85 reacts with most APMV-1, whereas the Mab 617/161 reacts only with PPMV-1 isolates within the APMV-1 group by HI [39]. Thus, we further characterised the P/Aus/3/11 isolate and 24 APMV-1 strains of different virulence with two Mabs 617/161 and U85, revealing that Mab 617/161 only reacts with the P/Aus/3/11isolate, which supported this finding. Additionally, the P/Aus/3/11 isolate showed extensive cross-reactions to classical NDV strains in the HI test with polyclonal antisera against the V4 and P/Aus/3/11 isolates. This indicates that the P/Aus/3/11 isolate is antigenically indistinguishable from classical APMV-1 strains using conventional HI with polyclonal sera.

The HA titre of PPMV-1 has been reported to differ from classical NDV strains, with the HA titre of PPMV-1 antigen used for the formulation of inactivated vaccine being lower than that of NDV strains La Sota and Ulster [40]. Conversely, some Italian PPMV-1 isolates had HA titres equal to or greater than 640, which was similar to the NDV strains La Sota and B1 [19]. In the current study, HA titres of the pigeon isolates mostly ranged from 2^6^ to 2^7^, with occasional titres of 2^8^ or 2^9^. Serial passage of the isolate in both embryos and CEFs did not significantly increase the HA titres. This is in agreement with the previous study that the HA activity was not affected by serial passage of PPMV-1 isolates in embryos [11].

One early study [41] showed that the period of hemagglutinin thermostability ranged from 30 to 120 min at 56 °C for the virulent strains of NDV tested and less than 5 min for lentogenic strains, suggesting that virulence of NDVs for chickens may be related to thermostability of the hemagglutinin. Further studies showed that virulence of NDVs for chickens was not related to thermostability of the hemagglutinin. Despite this, thermostability of the hemagglutinin of NDV has been used as a strain marker in epizootiologic studies [21]. Our results showed that the HA activity of the P/Aus/3/11 isolate was lost within 45 min at 56 °C, which was in agreement with reports of between 30 and 60 min [19], or 10 to 60 min [40]. As little information is available on HA thermostabilty of other PPMV-1 isolates, the possibility that HA thermostability could be used as a marker for virulence, or in epidemiological investigations merits further study.

The P/Aus/3/11 isolate had a slow elution rate from chicken red blood cells, which was consistent with that of some PPMV-1 strains [19]. The MDT results were also similar to other reports of pigeon isolates, with a range of nearly 90 hrs to as high as 160 hrs [19,31,42,43]. Therefore, these biological characteristics of the P/Aus/3/11 isolate were similar to those of other PPMV-1 strains.

Lentogenic NDV strains need the addition of exogenous trypsin to form syncytia in cell culture monolayers, whereas virulent strains do not [44]. Therefore, this property could be used to determine virulence of NDVs. It was suggested that the plaque size was directly related to virulence of NDVs [45]. However, other studies have shown that it cannot be considered as a reliable indicator for viral virulence as the plaque size highly depends on the use of certain viral mutants, strains and cell types [46,47]. Despite this, the plaque assay may be used to characterize NDV strains in modern ND research, particularly for recombinant NDV strains [48] due to it being less costly in comparison with live bird inoculation and it does not require the use of animals [49]. In the current study, the P/Aus/3/11 isolate and other reference NDV strains following infection of CEF cells were compared. The P/Aus/3/11 isolate formed plaques without exogenous trypsin and the plaque size was similar to a mesogenic reference isolate (Komarov), but slightly smaller than that of representative velogenic NDV isolates, whereas the lentogenic NDV V4 strain did not form plaques. This agrees with previous studies showing that NDV strains with avirulent F protein cleavage site motifs are not expected to form plaques on CEF without additional trypsin [36]. Based on the in vitro data, it is reasonable to speculate that the P/Aus/3/11 isolate appeared to be of intermediate virulence but is less virulent than the virulent NDVs used in the study.

Useful tests for the assessment of NDV virulence are the MDT in embryonated chicken eggs, the intravenous pathogenicity index (IVPI) in six-week-old chickens and the intracerebral pathogenicity index (ICPI) in one-day-old chickens. Besides providing a useful indication of virulence, MDT and IVPI are also considered to be sufficiently reliable, particularly for the assessment of NDV strains isolated from hosts other than chickens [50]. Currently, the OIE accepted methods to assess virulence of NDVs are either ICPI or the sequence analysis of the fusion protein cleavage site. The P/Aus/3/11 isolate displayed a ^112^RRQKRF^117^ motif at the fusion protein cleavage site, which is a typical motif for virulent NDV [44]. However, virulence of PPMV-1 does not always correlate with the cleavability of its fusion protein [51]. Due to the animal welfare implications of using the ICPI and IVPI tests, they were not conducted in this study, however, based on the in vitro data of virus growth in CEFs, plaque size, MDT and F protein cleavage site motif, the P/Aus/3/11 isolate seemed likely to be of mesogenic pathogenicity in chickens.

Phylogenetic analysis using complete fusion gene sequence revealed that P/Aus/3/11 and associated outbreak virus samples were most closely related to 2011 viruses from Belgium and China within genotype VI.2.1.1.2.2 but were not related to previous virulent NDVs responsible for ND outbreaks in chickens in Australia (Figure 2 and Figure S1). This indicates that the P/Aus/3/11 isolate, responsible for current outbreaks in pigeons, is likely to have arisen from the incursion of an exotic strain of NDV/PPMV-1. Genotype VI is the most diverse of all the NDV genotypes [8]. This is consistent with its viruses being derived from widespread columbid species and with the viruses being responsible for an ongoing panzootic since the 1980s. Interestingly, the Australian sequences analysed occupy a distinct clade within subgenotype VI.2.1.1.2.2, suggesting that the Australian strain evolved independently from the other European and Chinese viruses that belong to this group.

Reverse genetic studies have shown the importance of the viral replication complex in virulence [4,31,46]. The L-polymerase was shown to be associated with virulence following studies comparing the lentogenic La Sota strain and the mesogenic Beaudette C strain [4]. Similar studies between the PPMV-1 strain AV324-96 and the velogenic Herts 33 showed that the NP, P and L proteins were more active in Herts 33 than AV324 and played a significant role in virulence. Passaging the pigeon variant AV324 strain in chickens increased virulence and resulted in mutations in the virus replication complex. Virus replication was enhanced with residue changes, P-protein N37D, L-polymerase N1564S and V1694E [52]. None of these mutations were observed in the P/Aus/3/11 strain.

Previous studies showed that NDV strains isolated from other bird species may not indicate their potential virulence for chickens in standard pathogenicity tests unless the viruses were passaged several times in chickens [6,12,15]. This makes the risk assessment for transmission and pathogenicity to poultry difficult. In this study, inoculation of chickens with a high dose of the P/Aus/3/11 isolate did not produce any clinical signs of disease or indications of pathogenicity. However, it has previously been noted that a high challenge dose of PPMV-1, whilst not producing clinical signs, may induce viral shedding in chickens [32] and this is consistent with our findings which confirmed shedding from both the choana and cloaca (Table 3). Confirmation of infection was seen via seroconversion to P/Aus/3/11 in those birds that were sampled 3 weeks PI.

Given that virus shedding may occur with exposure of chickens to PPMV-1 (albeit at high doses), the possibility therefore exists that this virus may increase in pathogenicity with continued transmission and serial passage, as may occur in commercial poultry flocks housed in high densities. Therefore, PPMV-1 may still pose a significant risk to Australian poultry and further investigation of the potential pathogenicity of this virus is warranted.

## Figures and Tables

**Table 1:** Virus growth in chicken embryonated eggs.

Pass No.	Inoculum		Number of Dead Eggs Post Inoculation (PI)				Number of Live Eggs at the End of Culture
	Dilution	Egg No.	D2	D3	D4	D5	
1	NA	3		1 (0) *		1 (2)	1 (16) (C5 **)
2	neat	3		1 (0)	1 (16)	1 (64)	
3	10^−3^	21		1 (0)	12(16 ^†^), 6 (32 ^†^)		2 (64 ^†^) (C4)
4	10^−3^	36	7 (64 ^†^)	28 (128 ^†^)			1 (128) (C3)
5	10^−3^	6	1 (32)	3 (128 ^†^)	1 (128), 1 (256)		

* Number of eggs (HA titre); ** days post inoculation at the end of culture; ^†^ pooled allantoic fluids.

**Table 2:** Reactivity of monoclonal antibodies and polyclonal antisera with representative avian paramyxovirus serotype-1 (APMV-1) isolates by HI.

Isolates	Virulence	MAbs		Polyclonal Antisera	
		617/161	U85	P/Aus/3/11	V4
P/Aus/3/11	Unknown	1024	4	256	512
Texas GB	Neurotropic velogenic	<2	32	8	128
Herts 33	Viscerotropic velogenic	<2	16	16	512
Komarov	Mesogenic	<2	512	64	1024
Beaudette C	Mesogenic	<2	1024	64	1024
B1	Lentogenic	2	256	32	512
V4	Lentogenic	<2	4	32	1024

**Table 3:** HA-elution patterns of APMV-1 isolates.

Strain	Original HA Titre	HA-Elution after			Elution Pattern
		24 h	Resuspension	120 h	
P/Aus/3/11	128	32	128	<2	Slow
Texas GB	64	32	128	<2	Slow
Herts 33	128	8	256	<2	Slow
Komarov	256	256	512	<2	Slow
Beaudette C	256	64	128	32	Slow
B1	512	<2	<2	<2	Rapid
V4	256	256	512	256	Slow

**Table 4:** HA thermostability of the P/Aus/3/11 isolate at 56°C.

	Original HA Titre	Haemagglutinin Thermostability after (min)										
		1	5	10	15	20	25	30	45	60	90	120
P/Aus/3/11	128	128	64	64	64	32	32	8	<2	−		
Texas GB	64	64	32	32	32	16	16	16	4	4	<2	−
Herts 33	128	128	128	128	128	<2	<2	−				
Komarov	256	128	128	8	<2	−						
Beaudette C	256	256	256	128	64	64	64	64	64	64	32	32
B1	512	512	256	−								
V4	256	256	256	256	256	256	256	256	128	128	64	32

**Table 5:** Virus detection by real-time RT-PCR testing of cloacal and choanal swabs collected from SPF chickens experimentally infected with P/Aus/3/11 isolate.

Bird No.	Virus Detection (Ct) on Day Post Infection (Cloacal/Choanal Swabs)												
	1	2	3	4	5	6	7	8	9	10	12	14	21
**1**	-*/31.6 ^±^	-/34.8	-/36.9	-/36.7 ^x^									
**2**	-/34.0	35.9/34.9	-/31.2	-/33.6	-/35.2	-/-	-/-	-/-	-/-	-/-	-/-	-/38.1	ND
**3**	-/32.9	-/34.7 ^x^											
**4**	-/32.4	-/34.0	-/32.9	-/33.9 ^x^									
**5**	-/28.1	-/32.7	-/29.9	-/28.4	-/30.6	-/-	36.9/-	37.7/-	-/-	36.2/-	34.2/-	33.3/-	-/-
**6**	-/29.4	-/34.3	-/33.5	36.7/28.1	-/35.6	-/37.7 ^x^							
**7**	-/29.5	-/34.4 ^x^											
**8**	-/27.4	-/32.8	-/32.4	-/33.3	-/31.9	-/32.9	-/30.6	-/37.4 ^x^					
**9**	-/32.5	-/34.1	-/33.0	-/33.1	-/36.8	-/-	-/37.6	-/38.1	-/-	-/-	-/-	-/-	ND
**10**	-/33.3	-/31.8	-/35.9	-/33.5	-/31.7	-/36.5	36.8/-	33.4/34.8	33.5/36.7	31.7/36.6	38.0/-	-/-	ND
**11**	-/30.8	-/35.2	-/34.3	-/29.8	-/28.7	-/31.1 ^x^							
**12**	-/32.5	37.7/34.0	-/31.6	-/32.7	-/34.9	36.2/37.7	-/-	-/36.9	-/-	37.3/-	-/-	29.8/-	ND/-
**13**	-/30.4	-/31.0	37.6/33.5	-/29.2	-/33.2	35.6/37.7	-/37.0	36.9/− ^x^					
+ve%	0/100	15.4/100	9.1/100	9.1/100	0/100	22.2/66.7	28.6/42.9	42.9/57.1	20/20	60/20	40/0	60/20	ND

* realtime RT-PCR negative; ^±^ Realtime RT-PCR Ct value; ^x^ Humanely killed; ND, not done.

**Table 6:** Molecular characteristics of P/Aus/3/2011 PPMV-1 compared with closely related PPMV-1 strains and previous NDV isolated from Australia.

Strain	F Cleavage Site	HN-Extension (Length: Sequence)	Classification	Genbank Acc. No.
P/Aus/3/2011	RRQKRF	0	VI.2.1.1.2.2	MN462666
Aus-V4/66	GKQGRL	45: REARSSRLSQLREGWKDDIVSPIFCDAKNQTEYRRELESYAASWP	I.1.1	JX524203
Aus/98-1252	RRQRRF	9: REARSSRLS	I.1.1	AY935493
Belgium/11-08304	RRQKRF	0	VI.2.1.1.2.2	JX901123
China/BJ-2013	RRQKRF	0	VI.2.1.1.2.2	KJ808819

## Data Availability

The genome sequences generated in this study have been deposited into Genbank under accession numbers MN413534 to MN413538 and MN462663 to MN462668.

## Supplementary Materials

The following are available online at https://www.mdpi.com/1999-4915/13/3/429/s1, Figure S1: Maximum Likelihood (ML) tree.
