# Supplementary material for: In Vitro and In Vivo Characterization of a Pigeon Paramyxovirus Type 1 Isolated from Domestic Pigeons in Victoria, Australia 2011
Source: Viruses. 2021 Mar 8;13(3):429. doi: 10.3390/v13030429 (PMC7998256; doi:10.3390/v13030429)
Supplement: Supplementary file 1 [file viruses-13-00429-s001.zip › Supplementary figure 1 caption.docx]

Supplementary figure 1

Maximum Likelihood (ML) tree based on n=189 virus fusion protein gene sequences with the 2011 Australian PPMV-1 virus samples (denoted by ^) belonging in genotype VI.2.1.1.2.2 The P/Aus/3/11 isolate is indicated in red. Phylogenetic relationships were inferred using the ML method based on the General Time Reversible model (Nei and Kumar, 2000). Vertical lines to the right of tree denote NDV genotypes according to the nomenclature of Dimitrov et. al. (2019). Horizontal scale bar indicates the number of substitutions per site.
