# Supplementary figures and images for: In Vitro and In Vivo Characterization of a Pigeon Paramyxovirus Type 1 Isolated from Domestic Pigeons in Victoria, Australia 2011
Source: Viruses. 2021 Mar 8;13(3):429. doi: 10.3390/v13030429 (PMC7998256; doi:10.3390/v13030429)

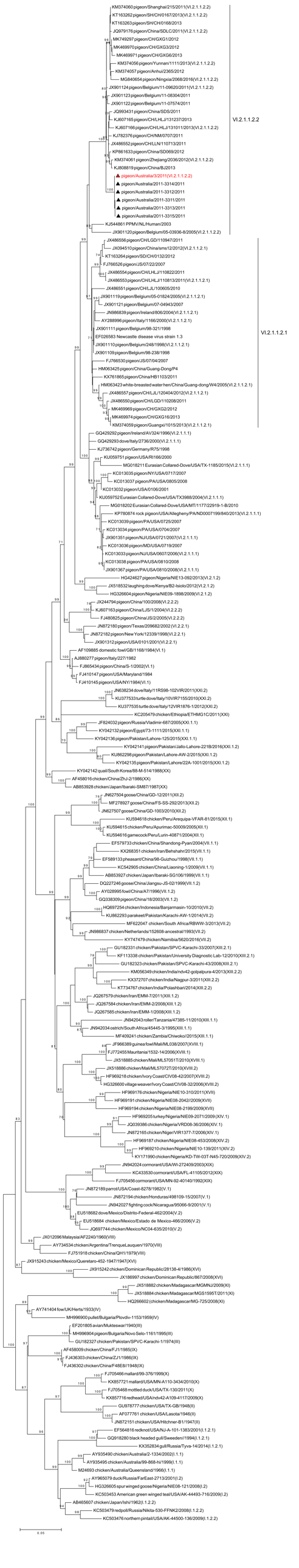

Supplement: Supplementary file 1 [file viruses-13-00429-s001.zip › Supplementary figure 1.tif]
